# The association between sociodemographic characteristics and dementia in patients with atrial fibrillation

**DOI:** 10.1007/s40520-019-01449-3

**Published:** 2020-01-11

**Authors:** Per Wändell, Axel C. Carlsson, Xinjun Li, Danijela Gasevic, Jan Sundquist, Kristina Sundquist

**Affiliations:** 1 Division of Family Medicine and Primary Care, Department of Neurobiology, Care Science and Society, Karolinska Institutet, Alfred Nobels Allé 12, 141 83 Huddinge, Sweden; 2 Center for Primary Health Care Research, Lund University, Malmö, Sweden; 3 Usher Institute of Population Health Sciences and Informatics, College of Medicine and Veterinary Medicine, University of Edinburgh, Edinburgh, UK; 4 School of Public Health and Preventive Medicine, Monash University, Melbourne, VIC Australia; 5 Department of Family Medicine and Community Health, Department of Population Health Science and Policy, Icahn School of Medicine at Mount Sinai, New York, USA; 6 Center for Community-based Healthcare Research and Education (CoHRE), Department of Functional Pathology, School of Medicine, Shimane University, Matsue, Japan

**Keywords:** Atrial fibrillation, Dementia, Gender, Socio-economic factors, Marital status

## Abstract

**Objectives:**

Association between socio-demographic factors and dementia risk is studied in general but not for atrial fibrillation (AF) patients.

**Methods:**

We studied AF patients ≥ 45 years in Sweden 1998–2012 (*n* = 537,513) using the Total Population Register for socio-demographic factors, the Swedish Cause of Death Register, and the National Patient Register (NPR) for incident dementia. Cox regression with hazard ratios (HR) and 95% confidence intervals (CI) was used for the association between exposure and outcome, adjusting for age and comorbidities.

**Results:**

Totally 30,332 patients (5.6%) were diagnosed with dementia during the follow-up (mean 5.4 years). Of these, 14,097 were men (4.9%) and 16,235 were women (6.5%). Lower educational levels (reference: highest level) were associated with increased dementia, HRs (95% CI) for basic school for men 1.23 (1.18–1.29) and women 1.36 (1.30–1.42), and middle-level school for men 1.17 (1.11–1.22) and women 1.28 (1.22–1.34). Divorced men and women (reference: married) showed increased risk of dementia, HR 1.07 (1.01–1.13) and 1.12 (1.06–1.18), respectively, while widowed men showed lower risk, HR 0.84 (0.80–0.88). High deprivation neighborhood socio-economic status (NSES; reference: medium level) was associated with increased dementia in men, HR 1.11 (1.05–1.17), and low deprivation neighborhood socio-economic status (NSES) with increased dementia in men and women, HR 1.12 (1.06–1.18) and 1.18 (1.12–1.24), respectively.

**Conclusions:**

Some results were expected, i.e. association between lower educational level and dementia. The higher risk of dementia in low deprivation NSES-areas could be due to a higher awareness about dementia, and subsequent earlier diagnosis and treatment of dementia.

**Electronic supplementary material:**

The online version of this article (10.1007/s40520-019-01449-3) contains supplementary material, which is available to authorized users.

## Introduction

Dementia is one of the greatest current global health challenges with substantial physical, social and economic impact on the lives of people living with the condition as well as on their family and society as a whole [1]. In 2015, it was estimated that there were approximately 47 million people worldwide living with dementia, and this number is expected to almost triple by 2050 with the increase mostly driven by an aging population [2]. In Sweden, there are approximately 150,000 people currently living with dementia (1.5%), and this figure is expected to increase by over 50% by 2050 [3]. These alarming statistics were one of the reasons for the Swedish Government to adopt a national strategy in 2018 for a more comprehensive approach to dementia care [4].

Many diseases are associated with an increased risk of dementia: cardiovascular diseases such as coronary heart disease (CHD) [5], atrial fibrillation [6, 7], stroke [8], but also non-vascular diseases such as diabetes [9, 10], and depression [11]. Atrial fibrillation (AF) is the most common arrhythmia in the world with a prevalence of 2% in Sweden [12]. Even if AF and dementia are more common in older adults, AF has also been associated with incident dementia among somewhat younger patients, e.g. below 67 years of age in one study [13]. Anticoagulant treatment in AF decreases the risk of both ischemic stroke and dementia [14], and consequently underuse of anticoagulants increases the dementia risk especially among older AF patients [15]. Besides, co-morbidities in AF, such as diabetes and depression, are also associated with a higher dementia risk [16, 17].

Regarding the association between individual socio-economic status (SES) and dementia, low individual SES has been found to predispose for dementia [18, 19]. However, age and medical co-morbidity seem to be more important factors that predispose for dementia than low SES. For area SES, a review found an association between community environment and cognitive function [20]. A French study found a higher risk of dementia among women but not among men living in deprived neighborhoods [21]. For marital status, a review found that being widowed or living lifelong single, but being divorced, showed a higher dementia risk compared to being married [22].

As regards patients with AF, it could be assumed, that the associations listed above are valid, but differences might also be present. In an earlier study of Swedish AF primary care patients, educational level and neighborhood SES were associated with increased risk of mortality, myocardial infarction and congestive heart failure, especially among men. However, for ischemic stroke, men with secondary schooling showed a higher risk compared to men with basic schooling [23]. Besides, most previous studies have studied the association between SES and prevalent but not incident dementia. Thus, some differences as regards AF patients and individuals in general in incident dementia could be at hand. A higher attention to early signs of dementia could be present, e.g. among siblings to patients, or among those being more educated or living in affluent neighborhood SES areas.

Therefore, the aim of the study is to describe the association between socio-economic status, both on individual level (educational level) and area level (neighborhood SES), and marital status with incident dementia among adult men and women with AF in Sweden.

## Methods

### Study population

The study population included all patients with diagnosed AF at hospitals, which can be detected in the National Patient Register (NPR) in Sweden, identified by the presence of the ICD-10 code (10th version of the WHO’s International Classification of Diseases) for atrial fibrillation (I48) from January 1, 1998 to December 31, 2012. The NPR includes data on in-patient diagnoses from 1998, and also on out-patients from 2001, from all Swedish hospitals. The Total Population Register and the Swedish Cause of Death Register were used for information on socio-demographic factors and mortality.

### Variables

As outcome variable, we studied the time from first AF diagnosis to first hospital diagnosis of dementia from January 1, 1998 to December 31, 2012. All diagnoses of dementia were included (with the following groups: F00 Alzheimer’s disease, F01 Vascular dementia, F02 Other dementia diseases, F03 Unspecified dementia, F10.7A Alcohol-related dementia, G30 Alzheimer). We excluded patients with any dementia diagnosis recorded prior to the first AF diagnosis.

Socio-demographic characteristics were defined according to the information found in the year of AF diagnosis in the individual patient. Age: Individuals were divided into the following age groups 45–54, 55–64, 65–74, 75–84 and ≥ 85 years of age. Individuals younger than 45 years were excluded. Sex: males and females. Educational level was categorized as ≤ 9 years (partial or complete compulsory schooling), 10–12 years (partial or complete secondary schooling) and > 12 years (college and/or university studies). Marital status was classified as married, unmarried, divorced or widowed. Region of residence: Individuals were classified as living in a large city, in Southern Sweden, or in Northern Sweden. Immigrant status was classified as born in Sweden or born in any other country [24].

Neighborhood socioeconomic status (NSES): The neighborhoods were derived from Small Area Market Statistics (SAMS). These were originally created for commercial purposes and pertain to small geographic areas with boundaries defined by homogenous types of buildings. The average population in each SAMS neighborhood is approximately 2000 people for Stockholm, and 1000 people for the rest of Sweden. The neighborhood index was derived from the following four variables: low educational status (< 10 years of formal education), low income (< 50% of the median individual income from all sources), unemployment and receipt of social welfare [25]. The neighborhood deprivation index was categorized into four groups: more than one standard deviation (SD) below the mean (low deprivation level or high NSES), more than one SD above the mean (high deprivation level or low NSES), and within one SD of the mean (moderate NSES or moderate deprivation level) used as reference group, and also unknown NSES, i.e. no data on neighborhood deprivation index were available. Missing data on NSES could be a sign of an unstable social situation.

We used the following diagnoses in the adjustments (for specific ICD-10 codes, please see Supplementary files): Hypertension, coronary heart disease (CHD), atrial fibrillation (AF), congestive heart failure (CHF), cerebrovascular diseases, obesity, diabetes mellitus, COPD, depression, anxiety disorders and alcoholism and related disorders [16, 26].

### Statistical analyses

Analyses were performed stratified by sex, showing counts and percentages for each variable. For baseline data Chi-2 analyses were performed separately by sex to examine differences between individuals with and without incident dementia.

The Cox regression models used the following variables in separate models for all dementia: age, all socio-demographic factors (educational level, marital status, neighborhood socio-economic status, region in Sweden and immigrant status), and all co-morbidities (hypertension, CHD, CHF, stroke, diabetes, obesity, COPD, depression, anxiety and alcoholism and alcohol-related diagnoses). Relevant co-morbidities were chosen according to findings in earlier articles [16, 17]. We also categorized the patients into those < 80 years of age, and ≥ 80 years of age, to get similar number of dementia cases in the two sub-groups. Pearson correlation coefficients were estimated between co-morbidities.

We also estimated the adjusted population attributable fraction (PAF), or population attributable risk (PAR) in percent for risk factors, as prevalence (%) among cases multiplied by HR-1/HR [27], using adjusted HRs for the different factors. PAF is useful in order to compare the effect of different risk factors on the incidence of the outcome.

A two-sided *p* value of *p* < 0.01 was considered statistically significant for variables in the *χ*^2^ analyses, and of < 0.05 in the Cox regression. All analyses were performed in STATA 15.2.

The study was approved by the regional ethics boards at Karolinska Institutet and Lund University.

## Results

In Table 1 we show the characteristics of the entire study population consisting of patients with AF (N = 537,513), stratified by sex (287,959 men and 249,554 women), and into those without or with a diagnosis of dementia. Mean follow-up was 5.4 years (sd 3.0), 5.6 years for men (sd 2.9) and 5.0 years for women (sd 3.0). Mean follow-up was higher among the youngest (men 8.0 and women 7.9 years), and lowest for the oldest (men 2.2 and women 2.4 years) (Supplementary Table 1). In total, 30,332 patients (5.6%) were diagnosed with dementia during follow-up, i.e. 14,097 men (4.9%) and 16,235 women (6.5%). Women were older (mean age 79.2 years, sd 10.1 years, vs. 73.4 years, sd 10.9 years), less likely to be married and more often widowed. There were statistically significant differences for most factors among men and women with or without incident dementia, with exceptions among men for diabetes and alcoholism, and among women for immigrant status, CHD and CHF.

**Table 1:** Baseline characteristics for patients aged ≥ 45 years with diagnoses of AF (*N *= 537,513), and with or without dementia in Sweden

	Men (*N* = 287,959)					Women (*N* = 249,554)				
	No dementia *N* = 273,862		Dementia *N* = 14,097		Diff	No dementia *N* = 233,319		Dementia *N = 16,235*		Diff
	Numbers	%	Numbers	%	*p* value	Numbers	%	Numbers	%	*p* value
Age groups (years)					< 0.001					< 0.001
45–54	18,517	6.8	75	0.5		6120	2.6	32	0.2	
55–64	47,740	17.4	717	5.1		20,465	8.8	351	2.2	
65–74	79,686	29.1	3594	25.5		50,093	21.5	2504	15.4	
75–79	45,362	16.6	3496	24.8		40,338	17.3	3583	22.1	
80–84	41,121	15.0	3438	24.4		47,076	20.2	4575	28.2	
≥ 85	41,436	15.1	2777	19.7		69,227	29.7	5190	32.0	
Educational level					< 0.001					< 0.001
≤ 9 years	122,078	44.6	6988	49.6		123,163	52.8	9443	58.2	
10–12 years	94,042	34.3	4463	31.7		66,360	28.4	4257	26.2	
> 12 years	57,742	21.1	2646	18.8		43,796	18.8	2535	15.6	
Marital status					< 0.001					< 0.001
Married	178,153	65.1	9293	65.9		99,758	42.8	5532	34.1	
Unmarried	28,176	10.3	915	6.5		13,982	6.0	859	5.3	
Divorced	35,542	13.0	1633	11.6		28,166	12.1	1877	11.6	
Widowed	31,991	11.7	2256	16.0		91,413	39.2	7967	49.1	
Region of residence					< 0.001					< 0.001
Large cities	144,895	52.9	7653	54.3		126,796	54.3	9409	58.0	
Southern Sweden	87,158	31.8	4226	30.0		72,401	31.0	4424	27.2	
Northern Sweden	41,809	15.3	2218	15.7		34,122	14.6	2402	14.8	
Immigrant status					< 0.001					0.05416
Born in Sweden	248,186	90.6	12,947	91.8		207,779	89.1	14,537	89.5	
Foreign Born	25,676	9.4	1150	8.2		25,540	10.9	1698	10.5	
Neighborhood deprivation level					< 0.001					< 0.001
Low	34,800	12.7	1722	12.2		24,354	10.4	1713	10.6	
Middle	134,552	49.1	6544	46.4		111,104	47.6	7160	44.1	
High	35,257	12.9	1775	12.6		33,684	14.4	2031	12.5	
Unknown	69,253	25.3	4056	28.8		64,177	27.5	5331	32.8	
Hospital Diagnoses										
Hypertension	114,409	41.8	5082	36.1	< 0.001	103,368	44.3	6344	39.1	< 0.001
Coronary heart disease	98,003	35.8	5278	37.4	< 0.001	69,936	30.0	5014	30.9	0.0145
Congestive heart failure	105,199	38.4	5692	40.4	< 0.001	92,230	39.5	6544	40.3	0.0499
Cerebrovascular diseases	64,431	23.5	5239	37.2	< 0.001	65,967	28.3	5557	34.2	< 0.001
Obesity	5360	2.0	77	0.5	< 0.001	4122	1.8	81	0.5	< 0.001
Diabetes mellitus	46,977	17.2	2414	17.1	0.9282	33,943	14.5	2185	13.5	< 0.001
COPD	31,122	11.4	1383	9.8	< 0.001	27,910	12.0	1476	9.1	< 0.001
Depression	8979	3.3	988	7.0	< 0.001	9835	4.2	1251	7.7	< 0.001
Anxiety	6011	2.2	408	2.9	< 0.001	7426	3.2	695	4.3	< 0.001
Alcoholism and related disorders	11,317	4.1	630	4.5	0.0506	2335	1.0	200	1.2	0.0045

The overall risk of dementia for men and women separately is shown in Table 2, and, in Table 3, divided by age-group, i.e. < 80 years of age, and ≥ 80 years of age. Regarding educational level, the risk for incident all-cause dementia was higher among those with the educational level of ≤ 9 years and 10-12 years, respectively, vs those with more than 12 years of education, however statistically non-significant for the educational level of 10-12 years for men and women < 80 years of age. Regarding marital status, being divorced was associated with higher dementia risk among men and women, however not among women ≥ 80 years of age. Among men, being unmarried or widowed was associated with a lower risk of dementia compared to being married, however only among those ≥ 80 years of age. Among women, being unmarried was associated with a higher risk in those < 80 years of age. As regards NSES for men, living in neighborhoods with high, low and unknown deprivation level was associated with a higher dementia risk compared to living in medium NSES deprivation level, however not statistically significant for men ≥ 80 years of age. As regards NSES for women, living in neighborhoods with high or unknown NSES was associated with a higher dementia risk compared to living in medium NSES deprivation level, while no increased risk was found for women living in high NSES deprivation level, although with a statistically borderline value for women < 80 years of age. There were no significant differences in the risk of dementia in the fully adjusted Cox regression models between men and women, and no significant interactions between gender and the socio-economic factors. Models for men and women combined, and also by dementia group are shown in Supplementary Table 2.

**Table 2:** Cox regression models (with hazard ratios (HRs) and 95% confidence interval (CI)) for incident hospital diagnosis of dementia among men and women in Sweden with diagnoses of AF and aged ≥ 45 years; patients with an earlier known hospital episode of dementia before AF diagnosis were excluded; Calculation of population attributable fraction (PAF) of socio-economic factors for incident dementia in men and women with atrial fibrillation

	Men					Women				
	Per cent	HR	95% CI	PAF (%)	95% CI	Percent	HR	95% CI	PAF (%)	95% CI
Educational level (> 12 years ref)										
≤ 9 years	49.6	**1.23**	**1.18–1.29**	**9.3**	**7.6; 11.2**	58.2	**1.36**	**1.30–1.42**	**15.4**	**13.4; 17.2**
10–12 years	31.7	**1.17**	**1.11–1.19**	**4.6**	**3.1; 5.7**	26.2	**1.28**	**1.22–1.34**	**5.7**	**4.7; 6.6**
Marital status (married ref)										
Unmarried	6.5	**0.88**	**0.82–0.94**	**– 0.9**	**− 1.4; -0.4**	5.3	1.03	0.96–1.11	0.2	− 0.2; 0.5
Divorced	11.6	**1.07**	**1.01–1.13**	**0.8**	**0.1; 1.3**	11.6	**1.12**	**1.06–1.18**	**1.2**	**0.7; 1.8**
Widowed	16.0	**0.84**	**0.80–0.88**	**– 3.0**	**− 4.0; -2.2**	49.1	1.01	0.97–1.05	0.5	− 1.5; 2.3
Neighbourhood deprivation level (medium ref)										
Low	12.2	**1.12**	**1.06–1.18**	**1.3**	**0.7; 1.9**	10.6	**1.18**	**1.12–1.24**	**1.6**	**1.1; 2.1**
High	12.6	**1.11**	**1.05–1.17**	**1.2**	**0.6; 1.8**	12.5	1.01	0.96–1.06	0.1	− 0.5; 0.7
Unknown	28.8	**1.67**	**1.60–1.76**	**11.6**	**10.8; 12.4**	32.8	**1.78**	**1.71–1.86**	**14.4**	**13.6; 15.2**

Models shown are adjusted for age, all other socio-demographic factors and co-morbidity. Bold values denote statistically significant values

**Table 3:** Cox regression models (with hazard ratios (HRs) and 95% confidence interval (CI)) for incident hospital diagnosis of dementia among men and women in Sweden with diagnoses of AF and aged ≥ 45 years, categorized by age-group; patients with an earlier known hospital episode of dementia before AF diagnosis were excluded

Variables	Men				Women			
	Aged 45–79 years (*n* = 7882)		Aged 80 + years (*n *= 6215)		Aged 45–79 years (*n* = 6470)		Aged 80 + yrs (*n* = 9765)	
	HR	95% CI	HR	95% CI	HR	95% CI	HR	95% CI
Educational level (> 12 years ref)								
≤ 9 yrs	**1.14**	**1.07–1.21**	**1.18**	**1.11–1.26**	**1.17**	**1.09–1.26**	**1.22**	**1.15–1.28**
10–12 yrs	1.07	1.00–1.14	**1.15**	**1.07–1.23**	1.06	0.98–1.14	**1.19**	**1.12–1.27**
Marital status (married ref)								
Unmarried	0.96	0.88–1.04	0.94	0.85–1.04	**1.13**	**1.02–1.26**	1.00	0.92–1.09
Divorced	**1.09**	**1.03–1.17**	**1.11**	**1.02–1.22**	**1.14**	**1.06–1.22**	1.04	0.91–1.12
Widowed	1.02	0.95–1.10	**0.89**	**0.84–0.94**	1.02	0.96–1.07	0.95	0.91–1.00
Neighbourhood deprivation level (medium ref)								
Low	**1.11**	**1.04–1.19**	1.08	0.99–1.16	**1.14**	**1.06–1.23**	**1.15**	**1.08–1.22**
High	**1.08**	**1.01–1.16**	**1.09**	**1.01–1.17**	1.07	0.99–1.15	0.99	0.93–1.05
Unknown	**1.65**	**1.55–1.75**	**1.61**	**1.51–1.72**	**1.82**	**1.70-1.95**	**1.65**	**1.57–1.74**

Bold values denote statistically significant values

When looking at mean follow-up, for individuals categorized by the educational levels it was 4.8 years (sd 3.1) for the lowest level (≤ 9 years), 5.7 years (sd 2.9) for the middle level (10–12 years) and 6.0 years (sd 2.8) for the highest level (> 12 years); for marital status 5.4 years (sd 3.2) for married and 5.3 (sd 2.7) for not married; for neighborhood deprivation for low level 6.2 years (sd 2.5), middle level 5.8 years (sd 2.6), high level 5.6 years (sd 2.7) and unknown level 4.0 years (sd 3.5).

We also estimated the adjusted population attributable fraction (PAF; Table 2). For men, lowest education level showed PAF 9.3%, and medium educational level 4.6%, with corresponding PAFs for women being 15.4% and 5.7%, respectively. For marital status and NSES, the PAFs showed low values, not exceeding 3%, except for living in unknown NSES (11.6% for men and 14.6% for women).

In the Pearson correlation matrix, the correlation coefficients showed low correlation between co-morbidities, with the highest correlation was between depression and anxiety, 0.26, and between CHD and congestive heart failure, 0.21 (Supplementary Table 3).

## Discussion

The findings of this study of AF patients include that lower educational levels were associated with higher risks of being diagnosed with incident dementia for both men and women. Regarding marital status, patterns were different among men and women, although with no significant interaction. Among men, a lower risk was found for being widowed, but for individuals being divorced a higher risk was found  among both men and women. As regards NSES deprivation level, for men, a higher risk of dementia was seen in both high and low deprivation NSES, but for women, only in low deprivation NSES level, however, with no significant interaction between gender. In addition, unknown deprivation NSES level was associated with higher risks in general among men and women.

Even if similarities with studies based on the general population could be expected, differences might also be at hand, as being diagnosed with a disease such as AF could possibly dilute associations with incident dementia. Being diagnosed with a disease such as AF could possibly decrease the differences, as AF is an important risk factor in itself for dementia.

Our findings concerning a higher dementia risk with lower educational levels among AF patients concur with earlier general findings [18, 19]. This well-known association between higher education and lower dementia risk could be due to different factors, among those the Cognitive Reserve (CR) hypothesis [28], explaining that individuals with a higher education, a higher IQ, or occupational attainment have lower risks of developing dementia, including Alzheimer’s disease [29], or vascular dementia. Besides, the PAFs showed rather high values, supporting the findings that lower educational level is an important risk for dementia also in patients with AF.

Our findings concerning marital status and incident dementia in AF patients are partly in line with, and partly different from, earlier findings in general. A review found that being married protected against dementia risk, while living lifelong single or being widowed showed an excess risk, and being divorced did not increase the risk [22]. An earlier Swedish study also found that living alone was associated with an increased dementia risk among both men and women compared to being married [30], while we found this to be true only for divorced men and women, and also for women < 80 years of age. In an earlier study of AF patients in primary care, a higher mortality among single living or divorced men were found, but not among women [23]. Regarding being unmarried or widowed, we found a lower risk of dementia among widowed men, in contrast to findings from a Taiwan study [31]. Our finding could possibly be a sign of not having siblings reacting to earlier signs of dementia among widowed men. This is also supported by the earlier mentioned review, where studies with clinical examinations for dementia showed a higher risk for unmarried compared to studies relying on register data [22]. However, the PAFs were on a low level in general, i.e. marital level only affected dementia risk marginally. Thus, the differences between AF patients in our study and individuals in general, i.e. concerning marital status, should be interpreted with some caution.

Our findings concerning the level of deprivation NSES and incident dementia are also partly in line with, and partly different from, what could be expected. In general, high deprivation SES is one of the strongest predictors of morbidity and also premature mortality in the world, even after consideration of the traditional risk factors for disease [32]. Lifestyle factors, attitudes and beliefs differ across SES levels, both for knowledge about the impact of different risk factors on health, and also for a lower probability of positive behavior changes [33]. Thus, there is an association with high deprivation NSES and many risk factors for diseases, including smoking, central obesity, physical inactivity and poor dietary habits, all of which are contributory factors to poor health [34], and also for cognitive decline and dementia [20, 35]. Besides, individuals living in low NSES are found to have lower use of anticoagulants [36], which is of importance as anticoagulants are shown to reduce incident dementia among AF patients [14, 37]. In addition, there are also psychological factors including feelings of inferiority and self-doubt among individuals residing in high deprivation SES neighborhoods [38]. In an earlier study of AF patients in primary care, a lower mortality was found for men living in low deprivation NSES, and a higher risk of myocardial infarction among men living in high deprivation HSES, while no such effect was found among women [23]. Thus, the findings of this study with a higher dementia risk among men living in deprived neighborhoods are what could be expected, while the higher risk among men and women living in low deprived neighborhoods contradicts what is expected. Besides, our findings are in contrast to a French study finding a higher risk of dementia among women but not among men living in high deprived neighborhoods [21]. One possible explanation for our finding among women could be that individuals living in low deprivation NSES are more concerned about their health, and seek care earlier for possible signs of dementia, with the possibility to get medical treatment, but this then could be true only for women. As dementia is a disease that often develops over many years, the time factor when reacting and seeking care could be of importance. However, the PAFs were on low levels, except for unknown NSES, why the differences between AF patients and individuals, in general, should be interpreted with some caution. The higher risk associated with unknown NSES deprivation level, on the other hand, is most likely a sign of a more unstable social situation, with an associated high rate of migration between different areas.

However, when looking at the studied factors among the AF patients in the present study, it must be emphasized, that there are also other factors of importance, such as leisure activities [39, 40], with one Swedish study finding that participating in leisure time activities, both physical, mental and social components, is associated with reduced risk of dementia [41], also when adjusting for age, educational level, co-morbidity, physical functioning and depressive symptoms. Stress is also a factor of importance for incident dementia [42, 43].

There are several limitations of this study. Dementia is frequently a disorder that develops over many years, and the diagnosis may be set earlier or later in the process. The follow-up time was rather short, especially among the oldest. Moreover, we have used data from registers, and the diagnoses could not be validated, and the categorization into different subgroups may be inaccurate, hence why we chose to focus on the total group of dementia. In general, the Swedish registers are shown to have high quality [44, 45]. However, in a review, studies based on clinical examinations for dementia showed a higher risk for unmarried individuals compared to studies relying on register data [22], thus indicating that studies based on register data underestimates the dementia risk. The findings may have been subject to residual confounding, such as leisure time activities, including social network, as these are found to be of importance [39, 41], and we had no access to such data. Besides, survival bias may have affected the results [46]. We used individual data for the patients at the time of first AF, and marital status, NSES and co-morbidities may have changed over time. Low socio-economic status, including low educational level [47], living in high deprivation NSES [48], and living alone [49, 50], is associated with higher mortality and lower life expectancy [32]. Moreover, AF could not be classified as paroxysmal, persistent or permanent and heart rhythm could not be classified as sinus rhythm or fibrillation rhythm. Another factor not being studied here is whether there are differences associated with adherence to drug prescriptions, e.g. to anticoagulants or other cardio-vascular drugs.

A major strength of this study was that we used national Swedish data, and that we were able to link clinical data from individual EPRs to data from national demographic and socioeconomic registers and that this data has less than 1% of information missing. Co-morbidities were selected as found in previous studies, and also from own earlier studies [16, 26].

Our findings do indicate that dementia diagnosis could be delayed in some groups, i.e. in individuals being divorced and in men living in higher deprivation areas. These findings suggest that physicians could be more active in suspecting and doing clinical investigations for dementia.

In conclusion, in this clinical setting with patients with AF, we found incident dementia was positively associated with lower educational levels among both men and women, and with being divorced among men and women, and also with high deprivation NSES level among men, as expected. Some findings were unexpected, such as the association among unmarried or widowed men with lower risk of dementia, and among men and women living in low deprivation NSES level. These findings could be due to an earlier awareness of signs of dementia in married individuals and lower deprivation NSES areas. The results must be interpreted with some caution, especially as the PAFs were low as regards marital status and NSES deprivation level, except for unknown NSES deprivation level, and they could have been affected by residual confounding or survival bias, or differences in the investigation and diagnosis of dementia in different socio-economic groups.

## Electronic supplementary material

Below is the link to the electronic supplementary material.

Supplementary material 1 (DOCX 43 kb)
