# Supplementary material for: The association between sociodemographic characteristics and dementia in patients with atrial fibrillation
Source: Aging Clin Exp Res. 2020 Jan 11;32(11):2319–27. doi: 10.1007/s40520-019-01449-3 (PMC7591421; doi:10.1007/s40520-019-01449-3)
Supplement: Supplementary file 1 — Supplementary material 1 (DOCX 43 kb) [file 40520_2019_1449_MOESM1_ESM.docx]

**Supplementary material**

*Co-morbidities with ICD-10 codes*

We identified the following cardiovascular co-morbidities from the EPRs among the individuals in the study population: hypertension (I10-15); coronary heart disease (CHD; I20-25), also including registered hospitalizations for myocardial infarction from the NPR; congestive heart failure (CHF; I50 or I110), also including hospitalizations for CHF from the NPR; cerebrovascular diseases (CVD; I60-69), also including registered hospitalizations for ischemic or haemorrhagic stroke from the NPR; diabetes mellitus (E10-14); obesity (E65-E68); COPD (J40-J47); depression (F32–F34, F38–F39); and anxiety disorders (F40–41).

| **Supplementary Table 1. Total person years and mean and median follow-up for patients aged ≥ 45 years with diagnoses of AF (*N*=537513).** | | | | | | | | | | | | |  |
| --- | --- | --- | --- | --- | --- | --- | --- | --- | --- | --- | --- | --- | --- |
|  | Men (N=287959) | | | | |  |  | Women (N=249554) | | | | |  |
|  | Total person years | Mean follow-up |  | Median follow-up | IQR |  |  | Total person years | Mean follow-up |  | Median follow-up | IQR | |
| Age groups (years) |  |  |  |  |  |  |  |  |  |  |  |  | |
| 45–54 | 142475 | 8.0 |  | 8 | 3-13 |  |  | 46412 | 7.9 |  | 7 | 3-12 | |
| 55–64 | 339343 | 7.3 |  | 7 | 3-11 |  |  | 147920 | 7.4 |  | 7 | 3-11 | |
| 65–74 | 469123 | 5.8 |  | 5 | 2-9 |  |  | 322301 | 6.3 |  | 5 | 2-10 | |
| 75–79 | 227668 | 4.8 |  | 4 | 1-7 |  |  | 231277 | 5.3 |  | 5 | 2-8 | |
| 80–84 | 158363 | 3.6 |  | 3 | 1-6 |  |  | 211641 | 4.1 |  | 3 | 1-6 | |
| ≥85 | 96264 | 2.2 |  | 1 | 0-3 |  |  | 178791 | 2.4 |  | 2 | 0-4 | |
| Educational level |  |  |  |  |  |  |  |  |  |  |  |  | |
| Compulsory schooling | 617280 | 4.9 |  | 4 | 1-8 |  |  | 593026 | 4.6 |  | 3 | 1-7 | |
| Secondary schooling | 508808 | 5.3 |  | 4 | 1-8 |  |  | 344236 | 5.0 |  | 4 | 1-8 | |
| College and/or university studies | 304240 | 5.3 |  | 4 | 1-8 |  |  | 197946 | 4.4 |  | 3 | 1-7 | |
| Marital status |  |  |  |  |  |  |  |  |  |  |  |  | |
| Married | 952439 | 5.9 |  | 5 | 2-9 |  |  | 482731 | 6.2 |  | 5 | 2-9 | |
| Unmarried | 144665 | 5.1 |  | 4 | 2-8 |  |  | 68754 | 4.7 |  | 4 | 2-7 | |
| Divorced | 194879 | 5.5 |  | 4 | 2-8 |  |  | 147493 | 5.1 |  | 4 | 2-8 | |
| Widowed | 140449 | 4.2 |  | 3 | 1-6 |  |  | 439108 | 4.5 |  | 3 | 2-6 | |
| Region of residence |  |  |  |  |  |  |  |  |  |  |  |  | |
| Large cities | 692919 | 4.7 |  | 3 | 1-8 |  |  | 555424 | 4.2 |  | 3 | 1-7 | |
| Southern Sweden | 502057 | 5.6 |  | 4 | 2-8 |  |  | 395220 | 5.2 |  | 4 | 2-8 | |
| Northern Sweden | 238260 | 5.5 |  | 4 | 2-8 |  |  | 187698 | 5.2 |  | 4 | 2-8 | |
| Immigrant status |  |  |  |  |  |  |  |  |  |  |  |  | |
| Born in Sweden | 1310170 | 5.1 |  | 4 | 1-8 |  |  | 1012871 | 4.6 |  | 3 | 1-7 | |
| Foreign Born | 123066 | 5.2 |  | 4 | 1-8 |  |  | 125471 | 4.9 |  | 4 | 1-8 | |
| Neighborhood deprivation |  |  |  |  |  |  |  |  |  |  |  |  | |
| Low | 203624 | 5.7 |  | 5 | 2-9 |  |  | 135611 | 5.3 |  | 4 | 2-8 | |
| Middle | 778546 | 5.6 |  | 5 | 2-8 |  |  | 615706 | 5.3 |  | 4 | 2-8 | |
| High | 182433 | 5.1 |  | 4 | 2-7 |  |  | 167639 | 4.8 |  | 4 | 2-7 | |
| Unknown | 268633 | 3.8 |  | 2 | 0-6 |  |  | 219386 | 3.2 |  | 1 | 0-5 | |
| Hospital Diagnoses |  |  |  |  |  |  |  |  |  |  |  |  | |
| Hypertension |  |  |  |  |  |  |  |  |  |  |  |  | |
| Non | 773154 | 4.7 |  | 3 | 1-7 |  |  | 580386 | 4.2 |  | 3 | 1-7 | |
| Yes | 660082 | 5.7 |  | 5 | 2-9 |  |  | 557956 | 5.2 |  | 4 | 2-8 | |
| Coronary heart disease |  |  |  |  |  |  |  |  |  |  |  |  | |
| Non | 895007 | 5.1 |  | 4 | 1-8 |  |  | 766253 | 4.5 |  | 3 | 1-7 | |
| Yes | 538229 | 5.1 |  | 4 | 1-8 |  |  | 372089 | 4.8 |  | 4 | 1-7 | |
| Congestive heart failure |  |  |  |  |  |  |  |  |  |  |  |  | |
| Non | 876867 | 5.1 |  | 4 | 1-8 |  |  | 667617 | 4.6 |  | 3 | 1-7 | |
| Yes | 556369 | 5.1 |  | 4 | 1-8 |  |  | 470725 | 4.6 |  | 4 | 1-7 | |
| Cerebrovascular diseases |  |  |  |  |  |  |  |  |  |  |  |  | |
| Non | 1083818 | 5.1 |  | 4 | 1-8 |  |  | 810744 | 4.6 |  | 3 | 1-7 | |
| Yes | 349418 | 5.1 |  | 4 | 1-8 |  |  | 327598 | 4.6 |  | 4 | 1-7 | |
| Obesity |  |  |  |  |  |  |  |  |  |  |  |  | |
| Non | 1402667 | 5.1 |  | 4 | 1-8 |  |  | 1115995 | 4.6 |  | 3 | 1-7 | |
| Yes | 30569 | 5.8 |  | 5 | 2-9 |  |  | 22347 | 5.5 |  | 4 | 2-8 | |
| Diabetes mellitus |  |  |  |  |  |  |  |  |  |  |  |  | |
| Non | 1170136 | 5.0 |  | 4 | 1-8 |  |  | 957593 | 4.6 |  | 3 | 1-7 | |
| Yes | 263100 | 5.5 |  | 4 | 2-8 |  |  | 180749 | 5.1 |  | 4 | 1-8 | |
| COPD |  |  |  |  |  |  |  |  |  |  |  |  | |
| Non | 1272604 | 5.1 |  | 4 | 1-8 |  |  | 999050 | 4.6 |  | 3 | 1-7 | |
| Yes | 160632 | 5.1 |  | 4 | 1-8 |  |  | 139292 | 4.9 |  | 4 | 1-7 | |
| Depression |  |  |  |  |  |  |  |  |  |  |  |  | |
| Non | 1380316 | 5.1 |  | 4 | 1-8 |  |  | 1083534 | 4.6 |  | 3 | 1-7 | |
| Yes | 52920 | 5.5 |  | 4 | 2-8 |  |  | 54808 | 5.1 |  | 4 | 1-8 | |
| Anxiety |  |  |  |  |  |  |  |  |  |  |  |  | |
| Non | 1398112 | 5.1 |  | 4 | 1-8 |  |  | 1095579 | 4.6 |  | 3 | 1-7 | |
| Yes | 35124 | 5.7 |  | 5 | 2-9 |  |  | 42763 | 5.4 |  | 4 | 2-8 | |
| Alcoholism and related disorders |  |  |  |  |  |  |  |  |  |  |  |  | |
| Non | 1373811 | 5.1 |  | 4 | 1-8 |  |  | 1127385 | 4.6 |  | 3 | 1-7 | |
| Yes | 59425 | 5.3 |  | 4 | 2-8 |  |  | 10957 | 4.6 |  | 4 | 1-7 | |

IQR denotes interquartile range

**Supplementary Table 2.** Cox regression models (with hazard ratios (HRs) and 95% confidence interval (CI)) for incident hospital diagnosis of dementia among patients aged ≥45 years with diagnoses of AF in Sweden; patients with an earlier known hospital episode of dementia before AF diagnosis excluded

|  | | | | | | | | | | | | | | | |
| --- | --- | --- | --- | --- | --- | --- | --- | --- | --- | --- | --- | --- | --- | --- | --- |
|  | Alzheimer's disease | | |  | Vascular dementia | | |  | Other dementia | | |  | All dementia | | |
|  | HR | 95% CI | |  | HR | 95% CI | |  | HR | 95% CI | |  | HR | 95% CI | |
| Males (ref. Females) | **0.89** | **0.85** | **0.94** |  | **1.20** | **1.14** | **1.26** |  | **0.95** | **0.92** | **0.99** |  | 1.00 | 0.97 | 1.02 |
| Educational level (ref. > 12 yrs) |  |  |  |  |  |  |  |  |  |  |  |  |  |  |  |
| ≤ 9 yrs | **1.17** | **1.10** | **1.25** |  | **1.25** | **1.17** | **1.33** |  | **1.43** | **1.36** | **1.49** |  | **1.30** | **1.26** | **1.35** |
| 10-12 yrs | **1.26** | **1.18** | **1.35** |  | **1.16** | **1.08** | **1.25** |  | **1.27** | **1.21** | **1.34** |  | **1.23** | **1.19** | **1.28** |
| Marital status (ref. Married) |  |  |  |  |  |  |  |  |  |  |  |  |  |  |  |
| Unmarried | **0.76** | **0.69** | **0.85** |  | **0.87** | **0.79** | **0.96** |  | 1.02 | 0.95 | 1.09 |  | **0.92** | **0.88** | **0.97** |
| Divorced | 0.98 | 0.91 | 1.06 |  | **1.11** | **1.03** | **1.20** |  | **1.09** | **1.03** | **1.15** |  | **1.07** | **1.03** | **1.11** |
| Widowed | **0.85** | **0.81** | **0.91** |  | **0.92** | **0.87** | **0.98** |  | **0.94** | **0.90** | **0.97** |  | **0.92** | **0.89** | **0.95** |
| Neighbourhood deprivation (ref. Middle level) |  |  |  |  |  |  |  |  |  |  |  |  |  |  |  |
| Low | **1.17** | **1.08** | **1.27** |  | **1.30** | **1.21** | **1.40** |  | **1.08** | **1.02** | **1.14** |  | **1.15** | **1.11** | **1.19** |
| High | 0.97 | 0.89 | 1.05 |  | 1.03 | 0.95 | 1.10 |  | **1.10** | **1.04** | **1.15** |  | **1.05** | **1.02** | **1.09** |
| Unknown | **3.12** | **2.92** | **3.33** |  | **1.59** | **1.49** | **1.69** |  | **1.31** | **1.25** | **1.37** |  | **1.72** | **1.67** | **1.78** |

Fully adjusted model, i.e. also by age, gender, region of residence, immigrant status and co-morbidity.

Bold values are statistically significant

**Supplementary Table x.** **Pearson Correlation Coefficients, (N = 537513). Prob > |r| under H0: Rho=0**

| **Pearson Correlation Coefficients, N = 537513  Prob > \|r\| under H0: Rho=0** | | | | | | | | | | | |
| --- | --- | --- | --- | --- | --- | --- | --- | --- | --- | --- | --- |
|  | **COPD** | **Alcoholism** | **CHD** | **Obesity** | **HT** | **Depression** | **CHF** | **Anxiety** | **Stroke** | **Diabetes** | **Outcome**  **Dementia** |
| **COPD** | \| 1.00000 \| \| --- \| \|  \| | \| 0.04120 \| \| --- \| \| <.0001 \| | \| 0.05773 \| \| --- \| \| <.0001 \| | \| 0.04089 \| \| --- \| \| <.0001 \| | \| 0.00485 \| \| --- \| \| 0.0004 \| | \| 0.02911 \| \| --- \| \| <.0001 \| | \| 0.11876 \| \| --- \| \| <.0001 \| | \| 0.04476 \| \| --- \| \| <.0001 \| | \| -0.02348 \| \| --- \| \| <.0001 \| | \| 0.02507 \| \| --- \| \| <.0001 \| | \| -0.01600 \| \| --- \| \| <.0001 \| |
| **Alcoholism** | \| 0.04120 \| \| --- \| \| <.0001 \| | \| 1.00000 \| \| --- \| \|  \| | \| -0.01940 \| \| --- \| \| <.0001 \| | \| 0.02366 \| \| --- \| \| <.0001 \| | \| 0.00836 \| \| --- \| \| <.0001 \| | \| 0.10399 \| \| --- \| \| <.0001 \| | \| -0.00973 \| \| --- \| \| <.0001 \| | \| 0.09776 \| \| --- \| \| <.0001 \| | \| -0.00969 \| \| --- \| \| <.0001 \| | \| 0.01473 \| \| --- \| \| <.0001 \| | \| 0.00064 \| \| --- \| \| 0.6409 \| |
| **CHD** | \| 0.05773 \| \| --- \| \| <.0001 \| | \| -0.01940 \| \| --- \| \| <.0001 \| | \| 1.00000 \| \| --- \| \|  \| | \| -0.00121 \| \| --- \| \| 0.3769 \| | \| 0.07232 \| \| --- \| \| <.0001 \| | \| 0.01198 \| \| --- \| \| <.0001 \| | \| 0.21125 \| \| --- \| \| <.0001 \| | \| 0.02309 \| \| --- \| \| <.0001 \| | \| 0.03922 \| \| --- \| \| <.0001 \| | \| 0.11294 \| \| --- \| \| <.0001 \| | \| 0.00401 \| \| --- \| \| 0.0033 \| |
| **Obesity** | \| 0.04089 \| \| --- \| \| <.0001 \| | \| 0.02366 \| \| --- \| \| <.0001 \| | \| -0.00121 \| \| --- \| \| 0.3769 \| | \| 1.00000 \| \| --- \| \|  \| | \| 0.06931 \| \| --- \| \| <.0001 \| | \| 0.02395 \| \| --- \| \| <.0001 \| | \| 0.01649 \| \| --- \| \| <.0001 \| | \| 0.02681 \| \| --- \| \| <.0001 \| | \| -0.03218 \| \| --- \| \| <.0001 \| | \| 0.09306 \| \| --- \| \| <.0001 \| | \| -0.02345 \| \| --- \| \| <.0001 \| |
| **HT** | \| 0.00485 \| \| --- \| \| 0.0004 \| | \| 0.00836 \| \| --- \| \| <.0001 \| | \| 0.07232 \| \| --- \| \| <.0001 \| | \| 0.06931 \| \| --- \| \| <.0001 \| | \| 1.00000 \| \| --- \| \|  \| | \| 0.01993 \| \| --- \| \| <.0001 \| | \| -0.00604 \| \| --- \| \| <.0001 \| | \| 0.03564 \| \| --- \| \| <.0001 \| | \| 0.03258 \| \| --- \| \| <.0001 \| | \| 0.09337 \| \| --- \| \| <.0001 \| | \| -0.02458 \| \| --- \| \| <.0001 \| |
| **Depression** | \| 0.02911 \| \| --- \| \| <.0001 \| | \| 0.10399 \| \| --- \| \| <.0001 \| | \| 0.01198 \| \| --- \| \| <.0001 \| | \| 0.02395 \| \| --- \| \| <.0001 \| | \| 0.01993 \| \| --- \| \| <.0001 \| | \| 1.00000 \| \| --- \| \|  \| | \| 0.00604 \| \| --- \| \| <.0001 \| | \| 0.25623 \| \| --- \| \| <.0001 \| | \| 0.01832 \| \| --- \| \| <.0001 \| | \| 0.00903 \| \| --- \| \| <.0001 \| | \| 0.04368 \| \| --- \| \| <.0001 \| |
| **CHF** | \| 0.11876 \| \| --- \| \| <.0001 \| | \| -0.00973 \| \| --- \| \| <.0001 \| | \| 0.21125 \| \| --- \| \| <.0001 \| | \| 0.01649 \| \| --- \| \| <.0001 \| | \| -0.00604 \| \| --- \| \| <.0001 \| | \| 0.00604 \| \| --- \| \| <.0001 \| | \| 1.00000 \| \| --- \| \|  \| | \| 0.00537 \| \| --- \| \| <.0001 \| | \| 0.00611 \| \| --- \| \| <.0001 \| | \| 0.10018 \| \| --- \| \| <.0001 \| | \| 0.00669 \| \| --- \| \| <.0001 \| |
| **Anxiety** | \| 0.04476 \| \| --- \| \| <.0001 \| | \| 0.09776 \| \| --- \| \| <.0001 \| | \| 0.02309 \| \| --- \| \| <.0001 \| | \| 0.02681 \| \| --- \| \| <.0001 \| | \| 0.03564 \| \| --- \| \| <.0001 \| | \| 0.25623 \| \| --- \| \| <.0001 \| | \| 0.00537 \| \| --- \| \| <.0001 \| | \| 1.00000 \| \| --- \| \|  \| | \| -0.00426 \| \| --- \| \| 0.0018 \| | \| 0.00717 \| \| --- \| \| <.0001 \| | \| 0.01404 \| \| --- \| \| <.0001 \| |
| **Stroke** | \| -0.02348 \| \| --- \| \| <.0001 \| | \| -0.00969 \| \| --- \| \| <.0001 \| | \| 0.03922 \| \| --- \| \| <.0001 \| | \| -0.03218 \| \| --- \| \| <.0001 \| | \| 0.03258 \| \| --- \| \| <.0001 \| | \| 0.01832 \| \| --- \| \| <.0001 \| | \| 0.00611 \| \| --- \| \| <.0001 \| | \| -0.00426 \| \| --- \| \| 0.0018 \| | \| 1.00000 \| \| --- \| \|  \| | \| 0.03754 \| \| --- \| \| <.0001 \| | \| 0.05182 \| \| --- \| \| <.0001 \| |
| **Diabetes** | \| 0.02507 \| \| --- \| \| <.0001 \| | \| 0.01473 \| \| --- \| \| <.0001 \| | \| 0.11294 \| \| --- \| \| <.0001 \| | \| 0.09306 \| \| --- \| \| <.0001 \| | \| 0.09337 \| \| --- \| \| <.0001 \| | \| 0.00903 \| \| --- \| \| <.0001 \| | \| 0.10018 \| \| --- \| \| <.0001 \| | \| 0.00717 \| \| --- \| \| <.0001 \| | \| 0.03754 \| \| --- \| \| <.0001 \| | \| 1.00000 \| \| --- \| \|  \| | \| -0.00500 \| \| --- \| \| 0.0002 \| |
| **Outcome**  **Dementia** | \| -0.01600 \| \| --- \| \| <.0001 \| | \| 0.00064 \| \| --- \| \| 0.6409 \| | \| 0.00401 \| \| --- \| \| 0.0033 \| | \| -0.02345 \| \| --- \| \| <.0001 \| | \| -0.02458 \| \| --- \| \| <.0001 \| | \| 0.04368 \| \| --- \| \| <.0001 \| | \| 0.00669 \| \| --- \| \| <.0001 \| | \| 0.01404 \| \| --- \| \| <.0001 \| | \| 0.05182 \| \| --- \| \| <.0001 \| | \| -0.00500 \| \| --- \| \| 0.0002 \| | \| 1.00000 \| \| --- \| \|  \| |

COPD denotes chronic obstructive pulmonary disease, CHD denotes coronary heart disease, HT denotes hypertension, CHF denotes congestive heart failure
